# Assessment of US Hospital Compliance With Regulations for Patients’ Requests for Medical Records

**DOI:** 10.1001/jamanetworkopen.2018.3014

**Published:** 2018-10-05

**Authors:** Carolyn T. Lye, Howard P. Forman, Ruiyi Gao, Jodi G. Daniel, Allen L. Hsiao, Marilyn K. Mann, Dave deBronkart, Hugo O. Campos, Harlan M. Krumholz

**Affiliations:** 1 Yale School of Medicine, New Haven, Connecticut; 2 Department of Radiology and Biomedical Imaging, Yale School of Medicine, New Haven, Connecticut; 3 Yale School of Management, New Haven, Connecticut; 4 Department of Health Policy and Management, Yale School of Public Health, New Haven, Connecticut; 5 Department of Economics, Yale College, New Haven, Connecticut; 6 Health Care Group, Crowell & Moring LLP, Washington, DC; 7 Department of Emergency Medicine, Yale School of Medicine, New Haven, Connecticut; 8 Department of Pediatrics, Yale School of Medicine, New Haven, Connecticut; 9 Yale New Haven Health System, New Haven, Connecticut; 10 Circulation: Cardiovascular Quality and Outcomes, Waltham, Massachusetts; 11 Society for Participatory Medicine, Newburyport, Massachusetts; 12 e-Patient Dave, LLC, Nashua, New Hampshire; 13 Stanford Medicine X, Stanford University School of Medicine, Stanford, California; 14 Patient-Oriented Scalable National Network for Effectiveness Research, University of California, San Diego, La Jolla; 15 California Precision Medicine Consortium, University of California, Davis, Sacramento; 16 Section of Cardiovascular Medicine, Department of Internal Medicine, Yale School of Medicine, New Haven, Connecticut; 17 Center for Outcomes Research and Evaluation, Yale–New Haven Hospital, New Haven, Connecticut

## Abstract

**Importance:**

Although federal law has long promoted patients’ access to their protected health information, this access remains limited. Previous studies have demonstrated some issues in requesting release of medical records, but, to date, there has been no comprehensive review of the challenges that exist in all aspects of the request process.

**Objective:**

To evaluate the current state of medical records request processes of US hospitals in terms of compliance with federal and state regulations and ease of patient access.

**Design, Setting, and Participants:**

A cross-sectional study of medical records request processes was conducted between August 1 and December 7, 2017, in 83 top-ranked US hospitals with independent medical records request processes and medical records departments reachable by telephone. Hospitals were ranked as the top 20 hospitals for each of the 16 adult specialties in the 2016-2017 *US News & World Report* Best Hospitals National Rankings.

**Exposures:**

Scripted interview with medical records departments in a single-blind, simulated patient experience.

**Main Outcomes and Measures:**

Requestable information (entire medical record, laboratory test results, medical history and results of physical examination, discharge summaries, consultation reports, physician orders, and other), formats of release (pick up in person, mail, fax, email, CD, and online patient portal), costs, and request processing times, identified on medical records release authorization forms and through telephone calls with medical records departments.

**Results:**

Among the 83 top-ranked US hospitals representing 29 states, there was discordance between information provided on authorization forms and that obtained from the simulated patient telephone calls in terms of requestable information, formats of release, and costs. On the forms, as few as 9 hospitals (11%) provided the option of selecting 1 of the categories of information and only 44 hospitals (53%) provided patients the option to acquire the entire medical record. On telephone calls, all 83 hospitals stated that they were able to release entire medical records to patients. There were discrepancies in information given in telephone calls vs on the forms between the formats hospitals stated that they could use to release information (69 [83%] vs 40 [48%] for pick up in person, 20 [24%] vs 14 [17%] for fax, 39 [47%] vs 27 [33%] for email, 55 [66%] vs 35 [42%] for CD, and 21 [25%] vs 33 [40%] for online patient portals), additionally demonstrating noncompliance with federal regulations in refusing to provide records in the format requested by the patient. There were 48 hospitals that had costs of release (as much as $541.50 for a 200-page record) above the federal recommendation of $6.50 for electronically maintained records. At least 6 of the hospitals (7%) were noncompliant with state requirements for processing times.

**Conclusions and Relevance:**

The study revealed that there are discrepancies in the information provided to patients regarding the medical records release processes and noncompliance with federal and state regulations and recommendations. Policies focused on improving patient access may require stricter enforcement to ensure more transparent and less burdensome medical records request processes for patients.

## Key Points

**Question:**

Are US hospitals compliant with federal and state regulations in their medical records request processes?

**Findings:**

This cross-sectional study of 83 US hospitals revealed that there was noncompliance with federal regulations for formats of release and state regulations for request processing times. In addition, there was discordance between information provided on medical records release authorization forms and that obtained directly from medical records departments regarding the medical records request processes.

**Meaning:**

Discrepancies in information provided to patients regarding medical records request processes and noncompliance with regulations appear to indicate the need for stricter enforcement of policies relating to patients’ access to their protected health information.

## Teaser

This cross-sectional study uses a simulated patient experience to evaluate the current state of medical records request processes of US hospitals in terms of compliance with federal and state regulations and ease of patient access.

## Introduction

The Privacy Rule under the Health Insurance Portability and Accountability Act of 1996 (HIPAA) gives patients the right of access to their protected health information.^1^ By federal regulation, medical record requests must be fulfilled within 30 days of receipt (with the possibility of a single 30-day extension) in the format requested by the patient if the records are readily producible in that format. Despite the establishment of the right of access and electronic health records becoming more widespread,^2,3,4^ patients may not be able to easily request, receive, and manage their medical records.^5,6^ Under guidance from the US Department of Health and Human Services, hospitals are permitted to impose a reasonable cost-based fee for the release of medical records, but costs still remain high.^7,8^ In addition, many hospitals add procedural obstacles that can limit patient access.^5^

With recent efforts by the federal government to launch the MyHealthEData initiative, which encourages patients to take control of their health data,^9^ it is important to assess and quantify the challenges that patients currently face in medical records request processes in the United States. We postulated that the subset of highly ranked hospitals in the United States would have request processes that are at least on par with the whole set of US hospitals. Thus, we focused our investigation on confirming full compliance with regulations related to requestable information, formats of release, costs of fulfilling requests, and processing times of requests in the top hospitals through a simulated patient experience.

## Methods

### Study Design and Population

We selected the top 20 hospitals for 16 different adult specialties in the 2016-2017 *US News & World Report* Best Hospitals National Rankings.^10^ Hospitals listed on multiple rankings, as well as hospital affiliates with the same medical records request process as their affiliated hospitals, were deduplicated from the study population. Medical records departments were telephoned to determine whether their request processes were separate from those of their affiliated hospital. This study was approved by the institutional review board as a not human research protocol at Yale University. The requirement of written informed consent and full disclosure was waived for this study. This study followed the Strengthening the Reporting of Observational Studies in Epidemiology (STROBE) reporting guideline.

In this cross-sectional study conducted between August 1 and December 7, 2017, we collected medical records release authorization forms from each hospital in the study population and subsequently telephoned each hospital’s medical records department to collect data on requestable information, formats of release, costs, and processing times using a predetermined script to minimize variation and biases across telephone calls (eFigure 1 in the Supplement). Information related to requesting records available on the webpage from which forms were downloaded was included as data collected with the authorization forms. Respondents to telephone calls were either employees of the medical records departments or representatives from an outsourced call center. A maximum of 5 attempts were made to reach each medical records department. A hospital was considered to be unreachable on each attempt if the telephone call was not answered, went to voice mail, or if the automated answering system did not allow the option to reach a representative. Thereafter, a voice message was left requesting a return telephone call. Seven days were allotted for a return telephone call; if no return telephone call was received, the hospital was classified as unreachable.

### Variable Definitions: Requestable Information, Formats of Release, Costs of Release, and Processing Times

We defined requestable information as information in either paper or electronic format residing within a health system that should consistently be associated with the medical record for all hospitals regardless of specialty and that could be requested through the general medical records request process (imaging and psychiatric records are often requested separately). Categories of requestable information included the entire medical record, laboratory test results, medical history and results of physical examination, discharge summaries, consultation reports, physician orders, and other. Paper formats of release included pick up in person, mail, and fax; electronic formats of release included email, CD, and online patient portal. If a form indicated electronic as a possible format of release without explicitly writing *email*, the format of release was inferred to be email if there was space to provide an email address. To qualify as being able to release records onto online patient portals by telephone call, the hospital must state that they can upload an entire medical record to their patient portal. Costs of release included any costs excluding shipping and postage. Processing times were mean times for processing medical records, if provided, or maximum times if a mean time was not disclosed. If asked which format of release would be requested to specify costs and processing times for a particular format, the standardized response was to request mailed records because mail is the only format of release present on all medical records release authorization forms.

### Comparative, Descriptive, and Narrative Analyses

We conducted data analyses for hospitals that were reachable by telephone. We compared data obtained from the authorization forms with data obtained from the telephone calls. Specifically, we calculated and compared the proportions of hospitals capable of releasing defined categories of information and in defined formats as elicited from the forms and from the telephone calls. The costs of release of records in paper formats were calculated based on the request of a hypothetical 200-page record. Costs elicited via telephone calls were compared with costs stated on the authorization forms, if any. Processing times were compared across all hospitals that provided mean times of release, grouped into the following categories: less than 7 days, 7 to 10 days, 11 to 20 days, 21 to 30 days, and more than 30 days. Mean processing times (if not available, then maximum processing times) were then individually compared with state requirements of hospitals.^11^ A complete comparison of costs and processing times for electronic formats of release of all hospitals in the study population was not conducted because not all hospitals release records electronically, electronic formats have varying costs, and many hospitals’ medical records departments reported not knowing the costs of some electronic formats.

We conducted narrative analyses of responses made by medical records department representatives during telephone calls, focusing on excluded information when requesting an entire medical record, possible formats of release, and reason for refusing release of select medical information and medical information in certain formats.

## Results

### Study Population Characteristics

We included 86 US hospitals in the study population after deduplication from an initial sample of 98 US hospitals. A total of 83 hospitals were reachable by telephone, with calls made between August 1 and December 7, 2017. Three hospitals were unreachable, 2 of which provided no option to leave a voice message or reach a department representative. Details of the 3 hospitals that were unreachable are included in the eAppendix of the Supplement. Thus, 83 hospitals, from 29 states, were included in our analysis (eFigure 2 in the Supplement).

### Requestable Information From Medical Records

**Figure 1.**
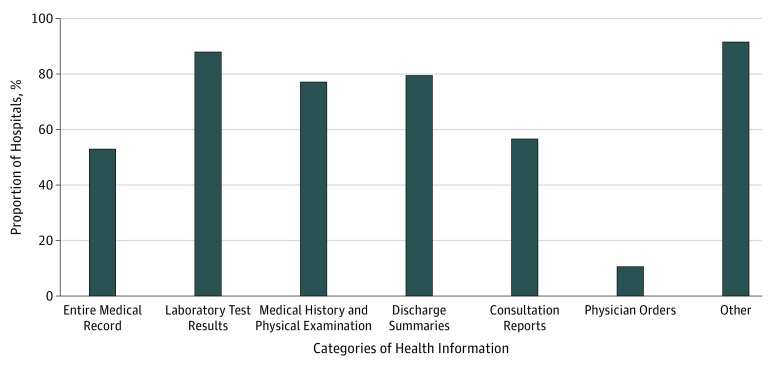
Proportion of Health Information Released by 83 Health Centers by Category of Health Information According to Options on Authorization Forms

Among the 83 hospitals, 44 (53%) provided patients the option on the forms to acquire their entire medical record. For individual categories of requestable information on the forms, as few as 9 hospitals (11%) provided the option of selecting release of physician orders and as many as 73 hospitals (88%) provided the option of selecting release of laboratory results. Most hospitals (76 [92%]) provided the option of an other category for requesting information not explicitly listed on the form (Figure 1).

Among the telephone calls, all the hospitals said they were able to release entire medical records to patients. When asked if any information would be withheld with a request of an entire medical record, 2 hospitals disclosed that nursing notes would not be released unless they were specifically requested. One hospital stated that selecting medical record abstract on the form would result in release of the entire medical record, whereas other hospitals communicated that an abbreviated medical record would be released.

### Formats of Medical Records Release

**Figure 2.**
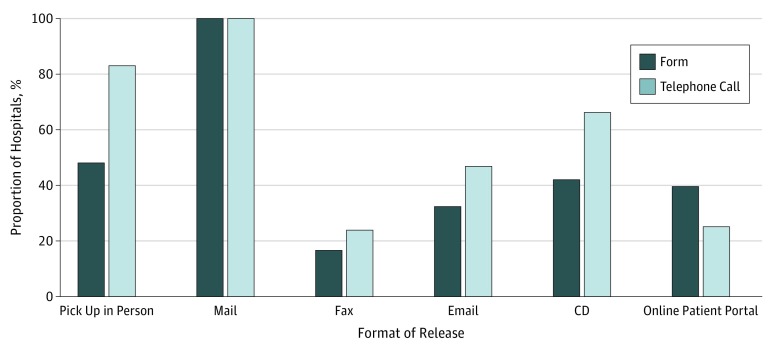
Comparison of Proportion of 83 Health Centers Releasing Records in Various Formats as Indicated on Authorization Form vs via Telephone Call

A greater number of hospitals stated in telephone calls vs on the forms that they were able to release information via the following formats of release: pick up in person (69 [83%] vs 40 [48%]), fax (20 [24%] vs 14 [17%]), email (39 [47%] vs 27 [33%]), and CD (55 [66%] vs 35 [42%]) (Figure 2). Fewer hospitals stated in telephone calls than on the forms that they were able to release information onto online patient portals (21 [25%] vs 33 [40%]). All hospitals stated in telephone calls and on the forms that they could release information via mail. Hospitals unable to provide records by fax stated that they could fax records only to physicians. Two hospitals reported not being able to release records electronically if the records were originally in a paper format.

### Costs of Medical Records Release

**Figure 3.**
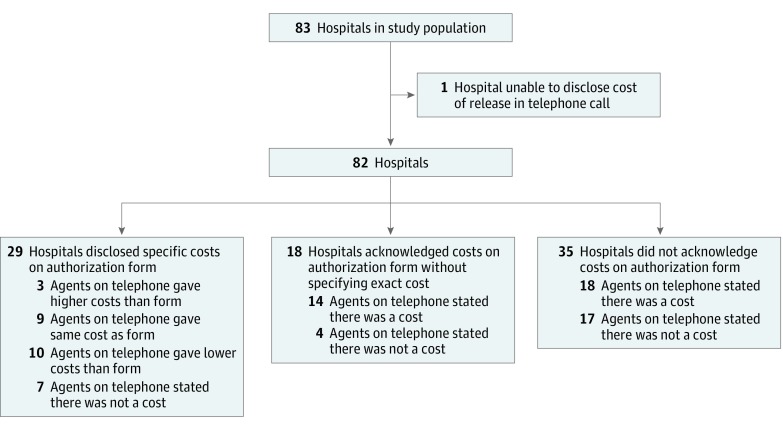
Comparison of Costs of Released Health Information Across the Aggregate Sample of 83 Health Centers by Authorization Form and by Telephone Call

On the authorization forms, 29 hospitals (35%) disclosed exact costs on the form or on the webpage from which the form was downloaded. One hospital stated on its form that it releases records free of charge, 18 (22%) disclosed that they would charge patients but did not specify a cost, and 36 (43%) did not specify any fees. For a 200-page record, the cost of release ranged from $0.00 to $281.54, based on the 29 hospitals that disclosed costs (Figure 3).

Among the telephone calls, 82 hospitals disclosed costs for paper formats of release and 1 hospital was unable to disclose costs of release, stating that costs are determined by an outside party. For a 200-page record, the cost of release as communicated in telephone calls ranged from $0.00 to $541.50. Of the 82 hospitals that disclosed costs, 48 hospitals (59%) stated costs of release above the federal recommendation of a $6.50 flat fee for electronically maintained records. Of the 29 hospitals that disclosed costs of release on their authorization form, 9 hospitals (31%) had the same fee schedule as that disclosed in the telephone calls, 10 (34%) had a less expensive fee schedule, 3 (10%) had a more expensive fee schedule, and 7 (24%) released records free of charge. Of the 18 hospitals that disclosed that they would charge patients without specifying a cost on the forms, 14 (78%) disclosed costs in the telephone calls, and 4 (22%) released medical records free of charge. Of the 35 hospitals that did not specify any costs on the forms, 18 (51%) disclosed costs in the telephone calls, and 17 (49%) stated that they released medical records free of charge (Figure 3).

For electronic formats of release, some hospitals reported charging $6.50, and some reported no charge for records released via an online patient portal. However, other hospitals charged the same fees for electronic formats and paper formats.

### Processing Times for Medical Records Release

**Figure 4.**
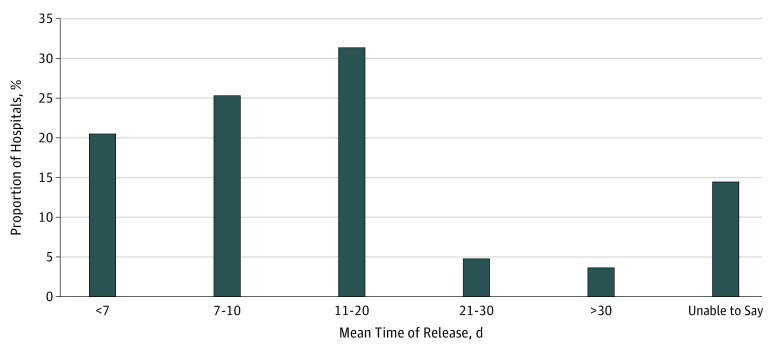
Comparison of Mean Time of Release of Records Across the Aggregate Sample of 83 Health Centers

Among the telephone calls, 71 hospitals provided mean times of release for paper copies of records. A maximum time of release was provided by 10 hospitals, and 2 hospitals were unable to specify a mean or maximum time of release. Of the hospitals that provided mean times of release, 17 (21%) reported mean times of less than 7 days, 21 (25%) in 7 to 10 days, 26 (31%) in 11 to 20 days, 4 (5%) in 21 to 30 days, and 3 (4%) in more than 30 days (Figure 4). In general, most hospitals were able to release records in electronic format in a shorter time frame than records in paper format.

The time of release for records in paper formats ranged from same-day release to 60 days. The time of release provided by each hospital was compared with its respective state’s access requirements (Table). Of the 81 hospitals that responded with times of release, 6 had ranges extending beyond their state’s requirement before applying the single 30-day extension granted by HIPAA.

**Table.** Compliance of Medical Records Request Processing Times With State Access Requirements^a^

State	State or HIPAA Requirement^b^	Hospital	Meets State Requirement
Alabama	Within 30 d from request	University of Alabama Hospital at Birmingham	Yes
Arizona	Within 30 d from request	Mayo Clinic Phoenix	Yes
		St. Joseph’s Hospital and Medical Center	Yes
California	Within 15 d from request	Cedars-Sinai Medical Center	Yes
		City of Hope	Yes
		Keck Medical Center of USC	Yes
		Rancho Los Amigos National Rehabilitation Center	Yes
		Scripps La Jolla Hospitals and Clinics	No; 4 wk or longer
		Stanford Health Care–Stanford Hospital	Yes
		UC Davis Medical Center	Yes
		UCLA Medical Center	Yes
		UC San Diego Medical Center–UC San Diego Health	Yes
		UCSF Medical Center	Yes
Colorado	Within 14 d from request	Craig Hospital	Yes
		National Jewish Health, Denver–University of Colorado Hospital, Aurora	Yes
Connecticut	Within 30 d from request	Hartford Hospital’s Institute for Living	Yes
		St. Francis Hospital	Yes
		Yale-New Haven Hospital	No; varies from 24 h to 46 d
Delaware	Within 30 d from request	Christiana Care–Christiana Hospital	Yes
Florida	Within 30 d from request	Bascom Palmer Eye Institute–Anne Bates Leach Eye Hospital	Yes
		Moffitt Cancer Center and Research Institute	Yes
		Mayo Clinic Jacksonville	Yes
		Tampa General Hospital	Yes
		University of Florida Health Shands Hospital	Yes
Georgia	Within 30 d from request	Emory University Hospital	Yes
		Shepherd Center	Yes
Illinois	Within 30 d from request	Rush University Medical Center	Yes
		Shirley Ryan AbilityLab	Yes
Iowa	Within 30 d from request	University of Iowa Hospitals and Clinics	Unknown
Kansas	Within 30 d from request	University of Kansas Hospital	Yes
Maryland	Within 21 d from request	Johns Hopkins Hospital	Yes
		Sheppard and Enoch Pratt Hospital	Yes
Massachusetts	Within 30 d from request	Austen Riggs Center	Yes
		Brigham and Women’s Hospital	Yes
		Dana Farber/Brigham and Women’s Cancer Center	Yes
		Massachusetts Eye and Ear Infirmary, Massachusetts General Hospital	Yes
		Massachusetts General Hospital	Yes
		McLean Hospital	Yes
		Spaulding Rehabilitation Hospital, Massachusetts General Hospital	Yes
Michigan	Within 30 d from request	Beaumont Hospital–Royal Oak	Yes
		Harper University Hospital	Yes
		University of Michigan Hospitals and Health Centers	No; up to 35 d
Minnesota	Within 30 d from request	Abbott Northwestern Hospital	Yes
		Mayo Clinic	Yes
Missouri	Within 30 d from request	Barnes–Jewish Hospital/Washington University	Yes
New Jersey	Within 30 d from request	Kessler Institute for Rehabilitation	Yes
New York	Within 30 d from request	Hospital for Joint Diseases, NYU Langone Medical Center	Yes
		Hospital for Special Surgery	Yes
		Long Island Jewish Medical Center	Yes
		Memorial Sloan Kettering Cancer Center	Yes
		Mount Sinai Hospital	Yes
		New York Eye and Ear Infirmary of Mount Sinai	Yes
		New York–Presbyterian University Hospital of Columbia and Cornell	Yes
		NYU Langone Medical Center	Yes
		St. Luke’s Hospital	Yes
North Carolina	Within 30 d from request	Duke University Hospital	Yes
		University of North Carolina Hospitals	Yes
		Wake Forest Baptist Medical Center	Yes
Ohio	Within 30 d from request	Cleveland Clinic	Yes
		Ohio State University Wexner Medical Center	No; 3-5 wk
Oklahoma	Within 30 d from request	Dean McGee Eye Institute, Oklahoma Medical Center	Yes
Oregon	Within 30 d from request	Oregon Health and Science University Hospital	Yes
Pennsylvania	Within 30 d from request	Hospitals of the University of Pennsylvania–Penn Presbyterian	No; typically 30 d, up to 60 d for older records
		Magee Rehabilitation Hospital	Yes
		MossRehab	Yes
		Rothman Institute at Thomas Jefferson University Hospital	Yes
		Thomas Jefferson University Hospital	Yes
		UPMC Presbyterian Shadyside	Yes
		Wills Eye Hospital, Thomas Jefferson University Hospital	Yes
South Carolina	Within 30 d from request	Medical University of South Carolina Medical Center	Yes
		Patewood Memorial Hospital	Yes
Tennessee	Within 30 d from request	Vanderbilt University Medical Center	Unknown
Texas	Within 15 d from request	Baylor University Medical Center	Yes
		The Heart Hospital Baylor Plano	Yes
		Houston Methodist Hospital	No; up to 30 d
		Menninger Clinic	Yes
		TIRR Memorial Hermann	Yes
		University of Texas MD Anderson Cancer Center	Yes
		University of Texas Southwestern Medical Center	Yes
Utah	Within 30 d from request	John A. Moran Eye Center, University of Utah Hospitals and Clinics	Yes
Washington	Within 15 d from request	Seattle Cancer Care Alliance/University of Washington Medical Center	Yes
		University of Washington Medical Center	Yes
Wisconsin	Within 30 d from request	University of Wisconsin Hospitals and Clinics	Yes

Abbreviations: HIPAA, Health Insurance Portability and Accountability Act; NYU, New York University; TIRR, The Institute for Rehabilitation and Research; UC, University of California; UCLA, University of California, Los Angeles; UCSF, University of California, San Francisco; UPMC, University of Pittsburgh Medical Center; USC, University of Southern California.

^a^ Hospitals labeled as not meeting state requirements do not necessarily defy legal requirements but do not promise to achieve the benchmark that is set by the state. A hospital being labeled as meeting state requirements for mean stated times of release does not preclude the possibility of the hospital taking longer than state requirements.

^b^ If state requirements are less strict than HIPAA requirements or give general timeframes, the 30-day requirement of HIPAA applies.

## Discussion

In our study of medical records request processes, we quantified the extent to which patients faced major barriers in obtaining their medical record data, and we identified areas in which a subset of US hospitals was noncompliant with federal and state regulations. We confirmed some of the challenges that patients face as described in a report released by the Office of the National Coordinator for Health Information Technology, such as long waiting periods and unclear request processes.^3^ Studies have surveyed health information management directors and privacy officers about patient access to personal health information,^6^ with 1 study focusing on the costs of obtaining records,^7^ but to our knowledge, no study has examined each aspect of the request process, from reviewing the authorization forms to calling medical records departments as a simulated patient. From our larger study sample of 83 hospitals in the United States, we investigated more closely the requestable information of medical records, formats of release, costs, and processing times and found that there were discrepancies between information relayed to patients through medical records release authorization forms and information given through telephone calls with medical records departments. Our findings in this simulated patient experience likely represent the best-case scenarios for these aspects of the request process because it seems unlikely that hospitals would make promises that they do not intend to fulfill.

There was a lack of transparency in the medical records request process. Only 53% of hospitals in the study sample explicitly stated on their authorization forms that they are capable of releasing entire medical records, when all the hospitals stated in the telephone calls that they could do so. Similarly, the possible formats of release on the forms did not match what was elicited through the telephone calls. Using the predetermined script for the telephone calls, we were able to clarify what records could be requested and how they can be requested. However, patients filling out authorization forms alone are often not presented with an accurate list of the records that they can request. Patients should not be expected to call medical records departments to find that parameters of the request process are different from those listed on the form. Although some hospitals were unwilling to release both paper and electronic records to patients, there are legal requirements under HIPAA to do so.^1^ The lack of a uniform procedure for requesting medical records across US hospitals highlights a systemic problem in complying with the right of access under HIPAA. Because every institution creates its own process and implements its own regulations, variability in what and how records can be received occurs.

Because 43% of hospitals did not reveal fee schedules on their authorization form or on the webpage from which the authorization form was obtained, patients were often not aware of the potential costs associated with requesting medical records. The Office for Civil Rights (a division of the US Department of Health and Human Services), which enforces HIPAA, recommends a flat fee of up to $6.50 for requests of electronically maintained records, a cost that is lower than many of the costs in our study, and states that per-page fees may not be charged for records maintained electronically, which was often the case for the hospitals in our study.^12^ In terms of processing times, at least 6 of the hospitals (7%) verbally reported processing times longer than the state-required time. Hospitals that provided mean processing times did not provide enough information to fully assess whether they were compliant with state requirements.

In our study, 2 of the 3 hospitals that could not be reached provided neither the option of speaking with a department representative nor the option of leaving a voice message. This practice impedes patients from gathering information that they may need to understand the medical records request process. Even for hospitals that were reachable, navigating through the automated voice response systems was often complicated before reaching a department representative.

Patients’ access to their medical records has long been proposed to benefit both patients and physicians.^13^ Studies have shown that patients want access to their records,^14^ and when patients have access, they have a better understanding of their health information, improved care coordination and communication with their physicians, and better adherence to treatment.^15,16,17,18^ With the Health Information Technology for Economic and Clinical Health Act of 2009 and its meaningful use criteria, adoption of electronic health records has become more widespread,^19,20,21,22,23^ but complicated, lengthy, and costly medical records request processes continue to inhibit patients from accessing their records. Recent policies are being implemented to further improve patient access, namely, the 2015 Health Information Technology Certification Criteria established by the US Department of Health and Human Services, which requires certified electronic health records to have application programming interfaces to enable patients to access and aggregate their information through innovative tools.^24^ The 21st Century Cures Act builds on the 2015 Health Information Technology Certification Criteria and sets the expectation that the US Department of Health and Human Services will promote a longitudinal health record.^25^

### Limitations

This study’s limitations largely stem from it having been conducted from the perspective of a single simulated patient, which may not represent all patients’ experiences. In this study design, telephone calls resulted in conversations with 1 individual at each hospital’s medical records department or its call center. This individual might disclose information not representative of the department or information conflicting with that given by other individuals in the department. Other individuals who contact the medical records departments with the same questions may receive different information, but our study could capture only 1 interaction with each health system. We know in the case of our own hospital (Yale-New Haven Hospital) that the official policy is different from what was reported in our telephone call. We contacted the 5 other hospitals with long processing times based on our calls and spoke with health information management directors from 3 of the hospitals (the other 2 did not respond to our email), all of whom reported that their official policies are different from what was reported to the simulated patient. In addition, our study design included only highly ranked US hospitals as part of the study population, which may or may not be representative of the medical records request process of all US hospitals. Future research is necessary to evaluate actual medical records requests made to a larger sample of US hospitals.

## Conclusions

Requesting medical records remains a complicated and burdensome process for patients despite policy efforts and regulation to make medical records more readily available to patients. Our results revealed inconsistencies in information provided by medical records authorization forms and by medical records departments in select US hospitals, as well as potentially unaffordable costs and processing times that were not compliant with federal regulations. As legislation, including the recent 21st Century Cures Act, and government-wide initiatives like MyHealthEData continue to stipulate improvements in patient access to medical records, attention to the most obvious barriers should be paramount.^9,25^

## Notes

Supplement. **eFigure 1.** Script for Calling Medical Records Departments to Elicit Information Regarding Process of Requesting Medical Records From the Patient Perspective **eFigure 2.** Schematic of Inclusion and Exclusion Criteria of Health Centers Included in Sample Population **eAppendix.** Results
