# Incorrect Data in the Abstract, Results, Discussion, Limitations, and Table

**DOI:** 10.1001/jamanetworkopen.2018.6463

**Published:** 2018-12-07

In the Original Investigation titled “Assessment of US Hospital Compliance With Regulations for Patients’ Requests for Medical Records,”^1^ published October 5, 2018, there were 6 hospitals (7%), not 7 (8%), that were noncompliant with state requirements for processing times. This error occurred in the last sentence of the Abstract Results, the last sentence of the Results, the third paragraph of the Discussion section, and the Limitations section. In the row for Colorado in the Table, the state requirement was changed to 14 days, and the state requirement was met by Craig Hospital. This article was corrected online.^1^
