# Supplementary material for: Assessment of US Hospital Compliance With Regulations for Patients’ Requests for Medical Records
Source: JAMA Netw Open. 2018 Oct 5;1(6):e183014. doi: 10.1001/jamanetworkopen.2018.3014 (PMC6324595; doi:10.1001/jamanetworkopen.2018.3014)
Supplement: Supplement. — eFigure 1. Script for Calling Medical Records Departments to Elicit Information Regarding Process of Requesting Medical Records From the Patient Perspective eFigure 2. Schematic of Inclusion and Exclusion Criteria of Health Centers Included in Sample Population eAppendix. Results [file jamanetwopen-1-e183014-s001.pdf]

Supplementary Online Content

Lye CT, Forman HP, Gao R, et al. Assessment of US Hospital Compliance With Regulations for Patients' Requests for Medical Records. *JAMA Netw Open.* 2018;1(6):e183014. doi:10.1001/jamanetworkopen.2018.3014

eFigure 1. Script for Calling Medical Records Departments to Elicit Information Regarding Process of Requesting Medical Records From the Patient Perspective

eFigure 2. Schematic of Inclusion and Exclusion Criteria of Health Centers Included in Sample Population

eAppendix. Results

This supplementary material has been provided by the authors to give readers additional information about their work.

© 2018 Lye CT et al. *JAMA Network Open*.

eFigure 1. Script for Calling Medical Records Departments to Elicit Information Regarding Process of Requesting Medical Records From the Patient Perspective

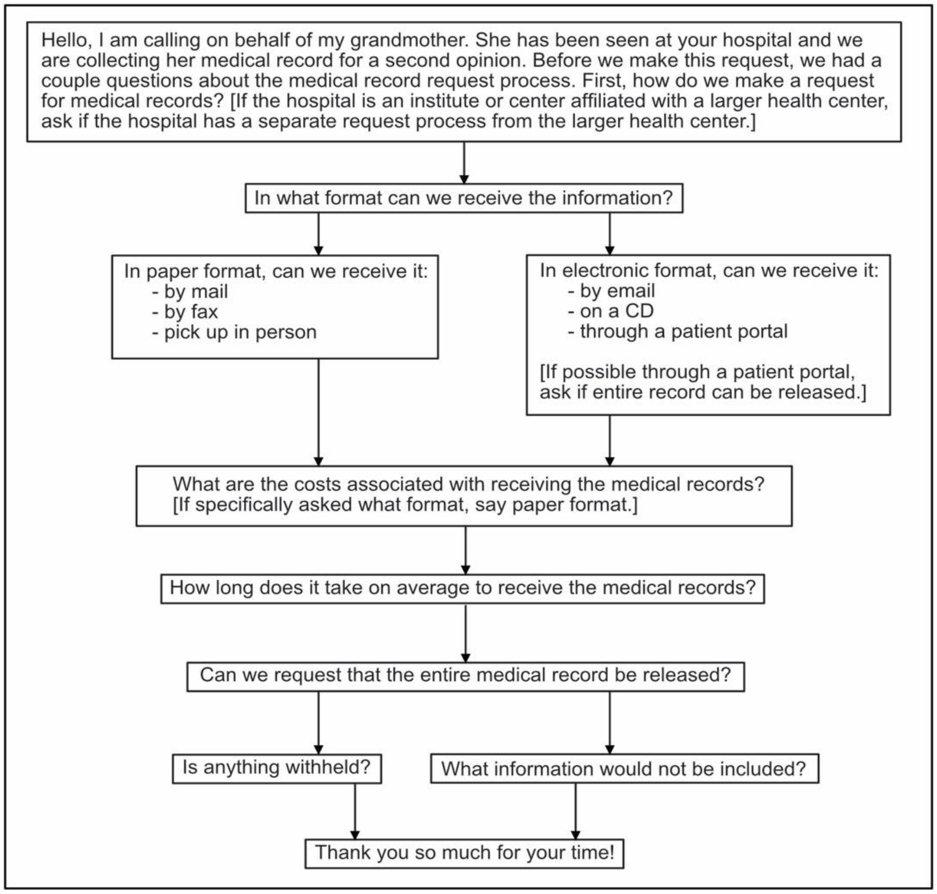

© 2018 Lye CT et al. *JAMA Network Open*.

eFigure 2. Schematic of Inclusion and Exclusion Criteria of Health Centers Included in Sample Population

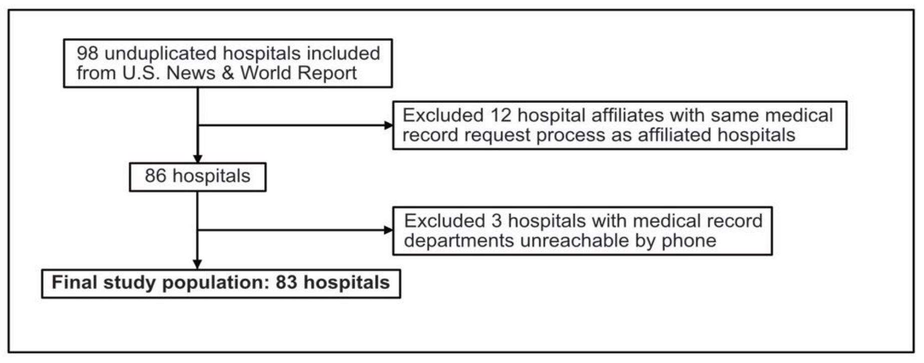

© 2018 Lye CT et al. *JAMA Network Open*.

eAppendix. Results

Study Population Characteristics

Hospitals in our study were classified as “unreachable” if on each attempt to call the hospital (up to five attempts were made), the phone call was not answered, went to voice mail, or if the automated answering system did not allow the option to reach a representative with whom to speak. Two hospitals, Northwestern Memorial Hospital and IU Health Academic Health Center, did not allow the option to leave a voice message or reach a department representative. Navigating through the automated answering system continued to loop back to the main menu. One hospital, the University of Colorado Hospital, made a return call after we left a voice message, but the call was not received on our end. We left another voice message, but received no return call from the hospital.

© 2018 Lye CT et al. *JAMA Network Open*.
